# Innovation in Data Visualisation for Public Policy Making

**DOI:** 10.1007/978-3-030-63693-7_4

**Published:** 2020-11-02

**Authors:** Paolo Raineri, Francesco Molinari

**Affiliations:** 1 DASTU, Politecnico di Milano, MILANO, Italy; 2 DASTU, Politecnico di Milano, MILANO, Italy; 3 Digitaal Vlaanderen, Brussels, Belgium; 4 Digitaal Vlaanderen, Brussels, Belgium; 5 Como, Italy; 6 Department of Architecture and Urban Studies, Politecnico di Milano, Milan, Italy

**Keywords:** Business intelligence, Technology innovation trends, Evidence-based policy making, Data scientist profession

## Abstract

In this contribution, we propose a reflection on the potential of data visualisation technologies for (informed) public policy making in a growingly complex and fast changing landscape—epitomized by the situation created after the outbreak of the Covid-19 pandemic. Based on the results of an online survey of more than 50 data scientists from all over the world, we highlight five application areas seeing the biggest needs for innovation according to the domain specialists. Our main argument is that we are facing a transformation of the business cases supporting the adoption and implementation of data visualisation methods and tools in government, which the conventional view of the value of Business Intelligence does not capture in full. Such evolution can drive a new wave of innovations that preserve (or restore) the human brain’s centrality in a decision making environment that is increasingly dominated—for good and bad—by artificial intelligence. Citizen science, design thinking, and accountability are mentioned as triggers of civic engagement and participation that can bring a community of “knowledge intermediaries” into the daily discussion on data supported policy making.

## Introduction: Data Visualisation Between Decision Support and Social Influence

Data visualisation is the art and science (Mahoney 2019) of graphically displaying large amounts of data in a visually attractive and simplified way, to facilitate understanding, decision and therefore action. This is done by a plethora of methods and tools, many of which—such as pie charts, dashboards, diagrams, infographics and maps—are quite familiar to those who have even basic notions of statistics or simply follow the news on traditional and social media. In fact, popularisation of data visualisation is a now well established phenomenon, which roughly materialised in the beginning of the new century, when tag clouds began to show up on blogs and websites and the so-called sparklines—very small graphs embedded in lines of journalistic text, to show up trends and variations—were invented.^1^

The effectiveness of using images instead of (too many) words to describe data has been evident to researchers from many disciplines, including both natural and social sciences.^2^ Even marketing—not to mention political communication—grasped the importance of visual displays to single out messages destined to be “digested” and transformed into actions by huge numbers of people, although sometimes at the cost of dissimulating, rather than refining, some true aspects of reality (Gonzalez 2019). In parallel, the so-called Business Intelligence field also took more and more benefit of visualisation technologies, especially with the growing size of data to be handled—both from within and outside the organisation—and the need to compress the decision making time of top and middle managers, by automating and simplifying the process of relevant information acquisition and analysis.

This peculiar aspect of data visualisation—being at the crossroad between decision support and social influence—has become particularly clear after the outbreak of the Covid-19 pandemic, when the first known cases of “deliberate censorship” have materialised on social media, such as Twitter and Facebook, to halt the spread of misinformation on how to protect against the virus. Not only are visuals now being used to place alerts on contested statements, but also the proliferation of infographics manipulating official data instrumentally has started to be cross-checked for reliability. In some extreme cases, Apple and Google are known to have removed some of these controversial apps from their stores (*Financial Express* 2020). On the other hand, the richness of mobility data as captured by the GPS of individual smart phones as well as the combination of textual contents with the geolocalisation of people interacting on social media have been widely perceived, maybe for the first time, as precious sources of information for decision making—and social influence again. Consider as examples: the use of Facebook and Google surveys done at Carnegie Mellon University to predict surges in the virus spread (Wired 2017); the Covid-19 Infodemics Observatory built at FBK in Trento^3^ using a global dataset of tweets and GPS information; and the business alliance “for the common good” between Apple and Google to enable interoperability between Android and iOS devices and jointly develop a Bluetooth-based contact tracing platform (Apple Newsroom 2020). The latter has generated, among others, the “Immuni” mobile app that is now widely advertised by the Italian government as a form of prevention against the unwanted effects of the “next wave” of contagion (Reuters 2020).

In this scenario, a crucial question to be posed to both researchers and practitioners of public administration, is whether we are facing the inauguration of a new trend for the take-up of data visualisation technologies in government. According to Fortune Business Insights (2020), the market of software applications for business intelligence and visual analytics, which nominally also includes public buyers, is estimated to hit $19.2 billion in the next seven years, from the current $8.85 billion, with an expected CAGR of 10.2% per. On the other hand, the pricing of business intelligence solutions is sometimes prohibitive, especially for small-sized public bodies and agencies, and statistics are missing on the impact of using open source solutions in the various application domains—such as public healthcare or urban planning. According to IDC (Shirer^2019^), the federal/central government share in the global market of business intelligence solutions is lower than 7% of total purchases. This figure either omits important buyers (e.g. local government or public utilities) and unpaid resources (such as free and open tools) or is simply an indication that the main business argument used to push adoption—“get to know more about what happens in your organisation, or just outside it, to take more informed decisions”—for a variety of reasons is not as compelling in the public sector as it seems to be for large corporations and medium sized enterprises.

This paper aims to stimulate a reflection in that direction, by asking the question of which kind of innovation is mostly needed to facilitate, rather than prevent, the take-up of data visualisation tools for public policy making. Answers to this question have been gathered from more than 50 domain experts (data scientists) from all over the world, by means of an online survey.^4^ After elaborating on received answers, we contrast this sort of indirect collection of user requirements with other emerging or growingly established technology trends—including e.g. Artificial Intelligence, IoT (Internet of Things), Edge Computing and AR/VR (Augmented/Virtual Reality). Our conclusion is that innovation in data visualisation may contribute to preserve a sort of demilitarised zone, where human decisions prevail over machine intelligence and initiatives. This aspect should be particularly appreciated by policy makers, but is curiously not well developed by specialised software vendors.

## Scoping the Experiences of Data Scientists

Among the several definitions of innovation we adopt the one by Loughlan (2016): “the pursuit of a better service or product that adds value to organizations, communities and to the wider society”. We asked domain experts from all over the world to help us define which value data visualisation tools (can) bring to public policy making. It turned out that there is not a single answer to such question. Value (to be) created depends very much on: (1) the policy maker’s goals, (2) if and how they are communicated to the data scientist, and (3) whether the latter properly understood them or not. This doesn’t mean that innovation is not part of the process—only that it must also involve the communication between interested parties. All professional data scientists agreed that the key rule to deliver an effective and useful visualisation for a policy making process is: know your “customer’s”^5^ goals. If the policy maker and the data scientists are not aligned on how the former will use the data provided by the latter, then the output of visualisation is at risk of being use-less.

As a complement to the above, it is relevant that data scientists identify the areas in which they see the biggest needs or pains from the perspective of their customers. In fact, one of the secure ways to create value with innovation is through tackling burning problems. The survey respondents mentioned the following issues:

- Multiple data source management: despite some recent progress in related technologies, data mash-up and cleaning are still the most time and resource consuming tasks of any data visualisation project. This is particularly true when multiple data sources are handled, lacking homogeneity and sometimes being non-standard, a frequent situation when working in/for the public sector.
- Rigorous data integration: the robustness and scientific lineage of the data used for policy making is vital. Unfortunately, it is still too difficult to certify and verify data sources and many steps forward should be done to avoid misinterpretation of what is being visualised. This pain has been reported by almost all of our survey respondents.
- Actionable information delivery: as obvious as it may be, data visualisation is supposed to generate ready-made insights that will lead the policy maker to a decision, speeding up the whole process and fostering stakeholders’ engagement. However, this is not always the case and of course, value creation is correspondingly reduced.
- Personalised user experience: the way different people look at a same dashboard or chart can be quite different. Yet as we mentioned above, data visualisation has (or should have) the capacity to trigger human brain to a specific decision and (re)action. In this perspective, we still know (and practice) too little on how to adjust the user experience according to the various personal behaviours while enjoying data.

These issues have been reported by more than 50 data visualisation experts, working with policy makers or public institutions in different countries. However, their relevance for the state of the art is also confirmed by the literature and personal experience highlights we are going to present below.

### Multiple Data Source Management

Various start-ups are tackling the issue of merging multiple data sources, but none of them seems to have reached the “nirvana” for the average data scientist. This is especially true for the non-relational sources. The main reason why the problem is not so easy to address is the variety of potential applications. Obviously, every process has its different goals, so the quality of your mash-up relies on what you should use those data for. This has been documented by Samuelsen, Chen and Wasson (2019) in a literature search of more than 115 publications, only restricted to the learning analytics domain. There are other reasons, however, which transform this issue into one of the hardest technical pains of this profession. Even if you use one of the good data wrangling tools now on the market, you still need at least some python/R basics or an old school manual on Excel to come up with a decent result. This means to allocate hundreds of working hours to simply get ready, instead of delivering the analytics and visualisation outputs.

### Rigorous Data Integration

This pain is both a “call for better data” and a request for improving their informative value. William Davies wrote a very good article in 2017 (Davies 2017) that still represents a good starting point for a crucial discussion to all of us. Getting scientifically validated data should always be a main concern for every policy maker, but when it comes to being sure about data lineage and research methods your legs start to crumble. Open data might be an alternative, but some problems remain: “1) Data is hard (or even impossible) to find online, 2) data is often not readily usable, 3) open licensing is rare practice and jeopardized by a lack of standards” (Lämmerhirt et al. 2017).

Data scientists also want to be sure that the visualisation output is not misleading in any way. In some cases, some colour palette mistakes, or naive data manipulations in the analytical steps, may lead to that very risky outcome, since the policy makers might ground their decision on a false representation of facts. In the future, Artificial Intelligence may help us solve this very tiny but insidious problem. Just as we now have language spell checkers for our weird grammar typos, some advisor bots may soon help us remember what we did with data and where it comes from, before taking any decision based on it.

### Actionable Information Delivery

Delivering an actionable output is in the top five rules of data scientists since Florence Nightingale taught to the world what the superpowers of data visualisation were meant for. She, as a nurse, drew herself data charts to boost decision making for the British army recovery in the Crimean war (Kopf 1916). But it’s not that easy. The very meaning of the word “actionable” puts a responsibility on data visualisation creators, especially within the domain of policy making. The challenge as we mentioned already, is to fulfil the policy maker’s goals while at the same time empowering him/her in a way that shortens the decision process time. Artificial Intelligence and particularly Natural Language Processing techniques are being trialled as alternative solutions for an actionable data visualisation. However, they still take too much processing time, resulting in a boring user experience and a poor quality of the generated insights.

### Personalised User Experience

Several authors (Toffler 1970; Davis 1987; Womack 1993; Anderson and Pine 1997) introduced the concept of mass customization as the “next big thing” in modern manufacturing. It took us maybe 30 years to reach a point of no return but since the 2000s it has become clear that the concept fits perfectly into actual human needs. We leave the moral question to other occasions. Here we simply note that a similar need is felt in the data visualisation for policy making community, as the responses to our survey demonstrated. We can imagine a visualisation able to adapt its colours, shapes, space, insights to personal behaviours and preferences. The fast-paced innovations of Machine Learning and more broadly Artificial Intelligence (also including mixed reality and face/voice recognition) are natural candidates to fulfil this requirement. This is probably the most attended innovation in data visualisation now, so we can hope to see it as a reality sooner than we think.

## A Critical Eye on Technology Innovation Trends

The oft-cited Artificial Intelligence is not the only route of innovation that can push up the threshold of data visualisation technologies in support of public decision making. Internet of things (IoT) (Sethi and Sarangi 2017) together with the new Edge Computing wave—the calculation model in which data is processed by the device itself or by a local computer or server, rather than being transmitted to a data centre (Premsankar et al. 2018), as well as voice/image recognition are also worth consideration.

IoT is the enabler of the “sensibility” of a country, region or city. We can say that IoT sensors act for a city just like the human receptors act for our body sensibility. That’s why MIT started to name smart city topics as the business of “sensible cities” (Dizikes 2016). The role of data visualisation in this context is quite obvious. The policy maker should merge sensible city projects, predictive analytics, and data visualisation to be able to act as the “wisdom brain” for good decisions.

Edge Computing is a distributed computing paradigm that brings computation and data storage closer to the location where it is needed, to improve response times and save bandwidth. This topic comes with high relevance in this paper because it’s already present in real world and only needs to be embraced to start producing effects. Endowed with this “data wisdom brain” the policy maker would boost exponentially his/her odds of success in every decision. Although such thoughts might lead to an enormous discussion about the future of humanity as a whole (Harari 2016) we could try to stay humble and admit that a more informed decision is always a better decision. The more data and information you have with you, the better your choices will be. Our assumptions and beliefs about the importance of this topic will become obvious if you agree with this sentence.

The same computational power needed to let IoT and predictive analytics play an effective role in decision making can also enable language-related and image-related technologies. There are many implications of this field. Voice recognition enables hands off interaction with machines. Natural language processing allows to understand human language shades and return warmer outputs. Image recognition enables to detect human emotions. The most famous implication of this kind of technologies is represented by the deep fake world (Vincent 2018). Here fake moving images of famous personalities are created leading the audience to believe in some weird videos (many about the presidents of USA, Russia, North Korea, Germany went viral in the social media just a few years ago). Notwithstanding the bad fame due to the heavy privacy implications, if a policy maker started to use these solutions then a new generation of data visualisation tools would help tremendously improve the engagement and truthfulness levels of the policy making cycle. This because that kind of technologies would speed up the creation of a lot of informative content and material and boost the engagement rate of the target audience thanks to a super personalized and customer-centric communication.

However, this potential does not seem to be perceived as such in the public policy making community. Let’s take another field as a benchmark case to machine learning, namely the professional basketball community. Not many years ago, M.I. Jordan commented that despite the diffused awareness of the importance of data analytics and therefore visualisation, “*we are no further ahead than we were with physics when Isaac Newton sat under his apple tree*” (Gomes 2014). And yet in the basketball community such knowledge gap was filled in by a single, although enthusiast, student of engineering with the simple (but brilliant) introduction of SOM techniques (Kohonen 1982, 2001) into players’ analytics (Bianchi et al. 2017). What can be the equivalent of that “connecting the dots” innovation in the policy making field? We have two or three possible ideas in mind.

A serious candidate is civic engagement. The ability to promote active interactions with citizens, not only as consumers but also producers of data, is nowadays well accepted as a wise and powerful way to procure useful information for public policy making. Getting granular, rigorously gathered data from a number of collaborative citizens is commonly called citizen science (Hand 2010; Castelvecchi 2016). This great way of engaging people with institutions has been used for an amazingly wide set of topics. But what is the link with data visualisation? Grasping the full potential of citizen science basically relies on people’s understanding of the data they collect. A great example is the CIESM JellyWatch project, a citizen science survey born after an overall jellyfish review in 2013 (Boero 2013) where a citizens mobile app enabled the collection of an enormous amount of data about Mediterranean Sea jellyfish distribution (Marshall 2010). Though bringing enormous benefits, citizen science must be managed in a good manner to avoid its risks. A good reading about the pros and cons of this approach is the article on Nature by Aisling Irwin (2018).

Another good candidate is the introduction of “design thinking” methodologies in the data visualisation journey. Design thinking is an umbrella term for the cognitive, strategic, and practical processes by which design concepts are developed. Many of the key processes of design thinking have been identified through studies, across different design domains, of cognition and activity in both laboratory and natural contexts. Design thinking is a way to put the end-user at the core of the design process. This does not only result in a faster and more effective output delivery for the policy maker, but introduces many connectors to the topic of citizens’ engagement. If policy makers would like to engage citizens nowadays, they should always look for a participation trigger. Data availability (and open data) draws an honest and transparent pathway that always acts as a nudge towards citizens’ engagement (citizen science plays a queen role in this game). Another nudge is to build the whole project with a “design thinking” vision. Citizens’ problems, their educational levels, interests, etc., everything should be taken into account. Communication with the audience should be tailored, direct and tackling only the main issues, avoiding the exchange of useless information. Following this train of logic, every data visualisation would appear familiar to the citizens, something made for them. However, in this quest for engagement “devil is in the details”. Both security and data quality issues play a major role in making this engagement pathway really workable for the policy maker who wants to benefit from the data visualisation features. The amazing work made by some projects in this area is already on the market waiting to be leveraged by public institutions (see Wired 2017; Kambatla et al. 2014). This is coherent with the digital transformation the whole world is having towards a human-centred approach to innovation (Kolko 2015).

Finally, choosing to visualise policy relevant data in a way that people both enjoy and understand is the perfect “final step” of an accountability process. Having a good and effective data visualisation leads to many positive implications: it highlights what is relevant and avoids distractions; proves the decision outcomes; helps to stay focused on budget and efforts; inspires hands-on participation; nurtures effective communication; flattens the learning curve on how to visualise data for decision making.

From a technological point of view there are some known tricks to make sure that data visualisation strikes the goals of civic engagement. The easiest way is always to start by keeping in mind the pre-attentive attributes like colours, shapes, movement, spatial positions etc. (Ware 2004). This could appear as a small issue, or a trivial aspect. But the more you push data visualisation forward, using it for real decision making, the more does this aspect become crucial, marking the difference between a good or a bad policy decision. Take the following example: you are in 2025 producing an important 3D visualisation directly going into the smart glasses of your citizens and forgot to think about colour-blind people. What could be the fallbacks? The number of delivered contents is also very important. This should be limited to those you want your citizens to follow. It is recommended giving to the users the possibility of drilling down and zooming in if they want, but the first look and feel must be lean and essential. Finally, it is important to always give to people the possibility of getting a “multi-dimensional” exploration of data. Maps are the best way to deliver a content in this way (and many of our survey contributors confirmed that).

## Conclusions and Way Forward

Be it because of the pains highlighted in our survey of domain experts or the technical limitations of many software applications, policy makers around the world do not seem to be ready yet to adopt data visualisation tools in support to decision making. And the main argument conventionally used to promote Business Intelligence in the private sector—that evidence is key to take informed decisions (Davies et al. 2000)—does not seem to work as well here.

In the previous section we have pointed at citizen science, design thinking and accountability as three triggers of civic engagement and participation that can bring a community of “knowledge intermediaries” into the daily discussion on policy making (Isett and Hicks 2019). This can help push that community ahead: from passively knowing the theory of a thing, to taking active action to carry it forward. But there is more: this evolution can drive a new wave of innovations preserving (or restoring) the human brain’s centrality in a decision making environment that is increasingly dominated—for good and bad—by Artificial Intelligence.

In fact, looking at “the big picture” it becomes clear that the ultimate goal (or outcome) of Artificial Intelligence is to prevent the beneficiaries of data visualisation from interacting directly with data. What we propose to do instead is to create visualisations that respect some particular constraints we gave them previously. Then we should look at the charts and try to infer a decision. By doing so we would always inject our human “bias” (for good and bad) into all the steps from database querying to the final chart design. Are we sure that we really want to get rid of this? Again, the answer may not be that straightforward.

Struggling with complexity may be a good argument in favour. As shown on the occasion of the Covid-19 health and information pandemics, there is no simple way other than visualisation to do justice of zillions of data growing in real time at an unprecedented speed. There is a large world of analytics and representation techniques to fight with complexity. The ability to process a big amount of data in various formats, from various sources, and deliver meaningful information to forecast future outputs to users is known as predictive analytics. Predictive analytics impacts almost every domain (Wang et al. 2018) and should be viewed as the main compass of every policy maker. The evolution of complex network analysis will deeply contribute to this area in helping to see both the big picture of a complex system and highlighting its peculiarities. We see this as the best way to be able to zoom in deeply to look for specific issues and solutions. Generally speaking, big data analytics open a bright future for our economies and societies (Amalina et al. 2020).

Keeping the control of our destinies—including the possibility of making mistakes—is a good reason against. Talking about the necessity of staying up to date, it is impossible to argue against the fact that nowadays we are all under pressure. Every public and private actor is somehow in a rush for the, so called, change. There is a shared pain about staying aligned with the world’s pace and being able to move fast and in the right direction at the same time. This feeling is even heavier for the policy makers that guard the keys of our world. But is this enough to decide that artificial intelligence should take full control?

Luckily, data science has evolved in parallel with the raising amount of data we produce every day. Although data scientists agree that the data amount grows faster than our ability to analyse them, we can say that “the challenge” is still fair enough. Therefore, leaving philosophical worries behind us, what we suggest to the data visualisation managers in the policy making area is to undertake an open and wide investigation to bring existing innovations from other sectors. Many great things are now ready to be implemented, coming from unexpected areas. Innovation in geo-spatial analytics developed for basketball could also be useful for smart mobility (Metulini et al. 2017). Old scientific research might lead to a solution for an institution’s data merging process (Buja et al. 1996). New methods and bio technologies (PHATE: Potential of Heat Diffusion for Affinity-based Transition Embedding) for visualising high-dimensional data (Moon et al. 2019) could revolutionize some aspects of the policy making cycle. Young students out in the world playing with data and “accidentally” solving many smart cities issues (Yang et al. 2019) could be scouted to speed up the innovation routines. Scientific projects already tailored for policy makers (Tachet et al. 2017) could be investigated with less fear. Future applications of mixed (i.e. augmented + virtual) reality (Joshi 2019) will be involved for sure in the next steps of data visualisation for policy making, with a particular attention to citizens’ engagement. Immersive videos, educational classrooms, policy maker meetings, political surveys, interactive discussions—the potentialities of mixed reality merged with data visualisations are infinite.

In conclusion, we can say that, once again in history, what will make the difference between remaining “stuck in the present” and evolving to a bright future will be the ability to contaminate mindsets and cross-fertilise domains, bearing in mind what really matters for people and avoiding to adhere to the “coolness-driven” purchasing decisions.

## Footnotes

1 https://en.wikipedia.org/wiki/Sparkline.

2 See: Data is beautiful: 10 of the best data visualization examples from history to today. https://www.tableau.com/learn/articles/best-beautiful-data-visualization-examples by Tableau Software (2020).

3 https://covid19obs.fbk.eu/#/.

4 The survey was a poll with five questions. Participants could answer using a free text form. The poll was done with Google Forms^TM^ and managed by the first author of this paper It was sent to members of the Data Visualization Society (https://www.datavisualizationsociety.com/). The audience was filtered before enabling access to the survey, to be sure about the participants’ background. All participants have worked for a public institution at least once as data scientists or data visualization managers. 52 of them answered the survey. They were asked if they wished to appear as supporters of this study, 4 of them answered positively and are acknowledged here: Alessandro Chessa, Evgeny Klochikhin, Luca Naso and Sevinc Rende. The survey was open for one month, from February 9th until March 9th 2020. The five questions were: (1) What are the 3 most important troubles you face while doing data visualization for a policy maker or for the public sector in general? (2) What are the 3 most important rules you follow to deliver a data visualization that is really useful for your client? (3) What kind of “visualization modality” do you prefer to engage citizens in producing data and be aware of them? (4) In your opinion, what kinds of innovation in data visualization are the most viable and feasible for the next future? (5) Any articles or book suggestion to know more about this topic? Any “talks” we must listen to? The 210 answers were transcribed and clustered in macro-topics. All of them were useful to act as foundation of this contribution.

5 The word “customer” in the present document is referred to policy makers being customers of data visualization.
